# Supplementary material for: Plastic Arbor: A modern simulation framework for synaptic plasticity—From single synapses to networks of morphological neurons
Source: PLoS Comput Biol. 2026 Feb 12;22(2):e1013926. doi: 10.1371/journal.pcbi.1013926 (PMC12900448; doi:10.1371/journal.pcbi.1013926)
Supplement: List of Legends of S1 Appendix — Fig A: More details on classical spike-timing dependent plasticity (STDP) and spike-driven homeostasis. Related to Fig 2 in the main article. Arbor implementations (in lighter blue) are cross-validated by comparison to Brian 2 (in orange) or theory. In (a–d), two Poisson spike sources stimulate an inhibitory and an excitatory synapse connecting to a single neuron (spikes are shown in red and blue, respectively). The excitatory connection undergoes STDP. (a) Membrane potential of the neuron (goodness of fit between the curves: CV=0.923, RMSE=1.265mV). (b) Conductance of the excitatory synapse (CV=0.996, RMSE=0.486μS). (c) Conductance of the inhibitory synapse (CV=0.997, RMSE=0.148μS). (d) Spike time mismatch measured by (tArbor−tBrian)/tBrian·100, where tArbor and tBrian are the postsynaptic spike times obtained from the Arbor and Brian 2 simulations, respectively. The result indicates that the difference in spike timing due to different implementations of the Poisson process is below 0.1%. (e) Classical STDP curve, compared with theoretical expectation Apre·exp(−Δt/τpre) for Δt>0 and Apost·exp(Δt/τpost) otherwise (CV>0.999, RMSE=0.001μS). (f) As opposed to Fig 2I in the main article, the resulting firing rate of the neuron in the absence of homeostatic plasticity is shown (CV=0.972, RMSE=1.674Hz). Fig B: Basic early- and late-phase plasticity dynamics with synaptic tagging and capture (STC), cross-validated with stand-alone simulator. Also note the the cross-validation with the stand-alone simulator in the main article (Fig 4) and with Brian 2 in Fig C. (a) Averaged noisy early-phase synaptic weight (see Eq 17) in the main article). Stimulating spikes reach the synapse at pre-defined times (indicated by bold gray arrows). Goodness of fit between the mean curves: CV=0.999, RMSE=0.040mV. (b) Limit cases of early- and late-phase synaptic weight (see Eq 17 and Eq 20 in the main article). The presynaptic neuron is stimulated with a strong current to spike at maximal rate [file pcbi.1013926.s001.pdf]

Appendix

—

Plastic Arbor: a modern simulation framework for synaptic plasticity – from single synapses to networks of morphological neurons

Jannik Luboeinski[1,2,3,✉], Sebastian Schmitt[1,2], Shirin Shafiee[1,2], Thorsten Hater[4], Fabian Bösch[5], Christian Tetzlaff[1,2,3]

[1] III. Institute of Physics – Biophysics, University of Göttingen, Göttingen, Germany
[2] Department for Neuro- and Sensory Physiology, University Medical Center Göttingen, Göttingen, Germany
[3] Campus Institute Data Science (CIDAS), Göttingen, Germany
[4] Jülich Supercomputing Centre, Forschungszentrum Jülich, Jülich, Germany
[5] Swiss National Supercomputing Centre, ETH Zürich, Zürich, Switzerland

✉ Correspondence: jannik.luboeinski@med.uni-goettingen.de

Main article:

10.1371/journal.pcbi.1013926

Simulator cross-validation – details at the synapse level

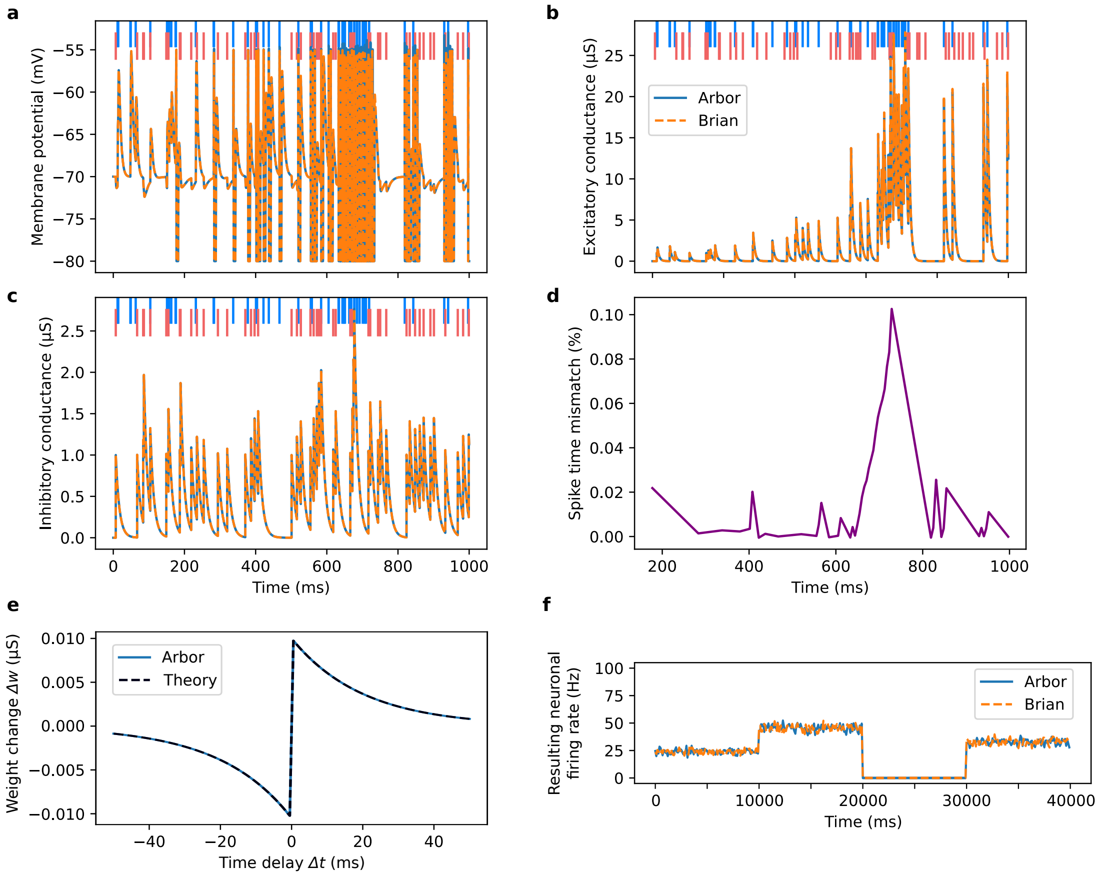

Fig A: **More details on classical spike-timing dependent plasticity (STDP) and spike-driven homeostasis.** Related to Fig 2 in the main article. Arbor implementations (in lighter blue) are cross-validated by comparison to Brian 2 (in orange) or theory. In (a–d), two Poisson spike sources stimulate an inhibitory and an excitatory synapse connecting to a single neuron (spikes are shown in red and blue, respectively). The excitatory connection undergoes STDP. **(a)** Membrane potential of the neuron (goodness of fit between the curves: CV = 0.923, RMSE = 1.265 mV). **(b)** Conductance of the excitatory synapse (CV = 0.996, RMSE = 0.486 µS). **(c)** Conductance of the inhibitory synapse (CV = 0.997, RMSE = 0.148 µS). **(d)** Spike time mismatch measured by $\left(t^{\text{Arbor}} - t^{\text{Brian}}\right)/t^{\text{Brian}} \cdot 100$, where t^{Arbor} and t^{Brian} are the postsynaptic spike times obtained from the Arbor and Brian 2 simulations, respectively. The result indicates that the difference in spike timing due to different implementations of the Poisson process is below 0.1%. **(e)** Classical STDP curve, compared with theoretical expectation $A_{\text{pre}} \cdot \exp(-\Delta t/\tau_{\text{pre}})$ for $\Delta t > 0$ and $A_{\text{post}} \cdot \exp(\Delta t/\tau_{\text{post}})$ otherwise (CV > 0.999, RMSE = 0.001 µS). **(f)** As opposed to Fig 2I in the main article, the resulting firing rate of the neuron in the absence of homeostatic plasticity is shown (CV = 0.972, RMSE = 1.674 Hz).

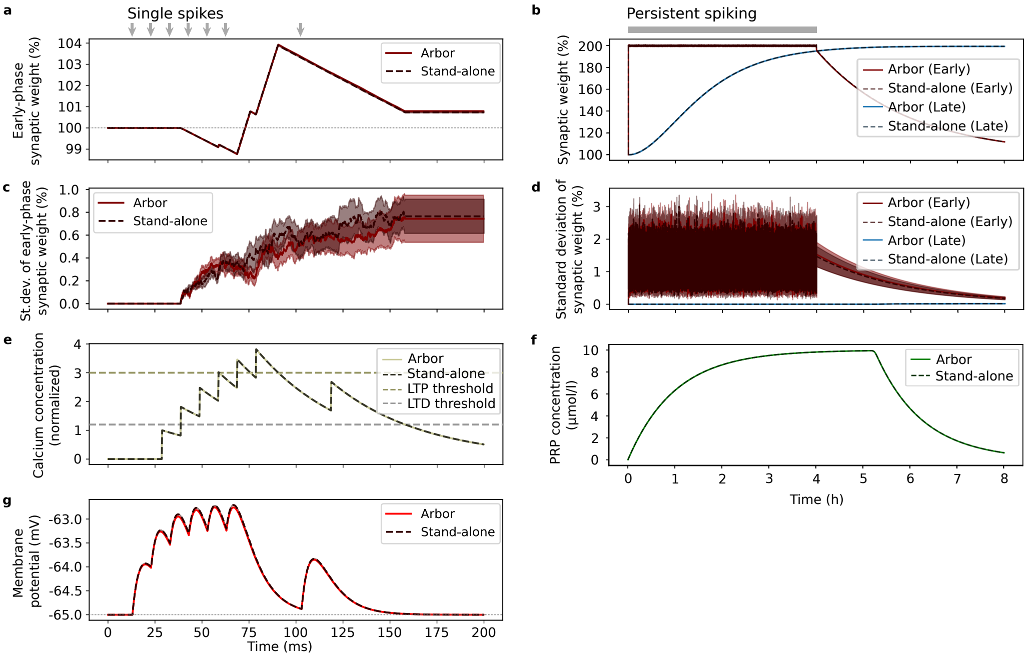

Fig B: **Basic early- and late-phase plasticity dynamics with synaptic tagging and capture (STC), cross-validated with stand-alone simulator.** Also note the the cross-validation with the stand-alone simulator in the main article (Fig 4) and with Brian 2 in Fig C. **(a)** Averaged noisy early-phase synaptic weight (see Eq. 17) in the main article). Stimulating spikes reach the synapse at predefined times (indicated by bold gray arrows). Goodness of fit between the mean curves: CV = 0.999, RMSE = 0.040 mV. **(b)** Limit cases of early- and late-phase synaptic weight (see Eq. 17 and Eq. 20 in the main article). The presynaptic neuron is stimulated with a strong current to spike at maximal rate (duration of the stimulation indicated by gray bar). The late-phase weight has been shifted for graphical reasons (also cf. Eq. 20; early phase: CV = 0.201, RMSE = 0.221 mV; late phase: CV $>$ 0.999, RMSE = 0.055 mV). **(c)** Standard deviation of the noisy early-phase synaptic weight (RMSE = 0.067 mV), and **(d)** standard deviation of early- and late-phase synaptic weight (early phase: RMSE = 0.133 mV; late phase: RMSE = 0.004 mV), demonstrating the matching of the stochastic properties of the two solvers. **(e)** Postsynaptic calcium concentration, which successively crosses the thresholds for depression (LTD) and potentiation (LTP) (cf. Eq. Eq. 19 in the main article; CV $>$ 0.999, RMSE = 0.065). **(f)** The postsynaptic PRP concentration rises until it reaches its maximum due to the continued stimulation (cf. Eq. 21 in the main article; CV = 0.998, RMSE = 0.107 µmol/l). **(g)** Membrane potential of the postsynaptic neuron (CV $>$ 0.999, RMSE = 0.151 mV). Continuous lines (specified in the legends) represent the dynamics simulated in Arbor. Darker, dashed lines represent the results of the stand-alone simulator [1]. Fine, dotted lines represent the baseline level of the respective quantity. Basic early-phase plasticity dynamics (a,c,e,g): average across 10 batches, each consisting of 100 trials. Basic late-phase plasticity dynamics (b,d,f): average across 10 batches, each consisting of 10 trials. The noise seeds were drawn independently for each trial. Error bands represent the standard error of the mean (often too small to be visible).

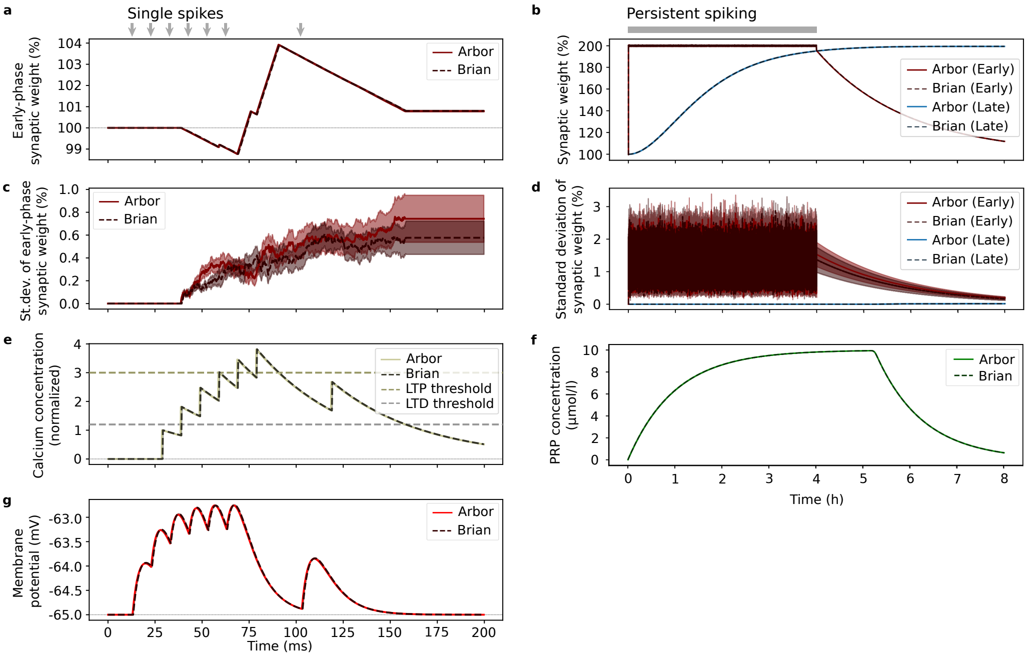

Fig C: **Basic early- and late-phase plasticity dynamics with synaptic tagging and capture (STC), cross-validated with the Brian 2 simulator.** Also see the the cross-validation with the stand-alone simulator in Fig B and in the main article (Fig 4). **(a)** Averaged noisy early-phase synaptic weight (see Eq. 17) in the main article). Stimulating spikes reach the synapse at pre-defined times (indicated by bold gray arrows). **(b)** Limit cases of early- and late-phase synaptic weight (see Eq. 17 and Eq. 20 in the main article). The presynaptic neuron is stimulated with a strong current to spike at maximal rate (duration of the stimulation indicated by gray bar). The late-phase weight has been shifted for graphical reasons (also cf. Eq. 20). **(c)** Standard deviation of the noisy early-phase synaptic weight, and **(d)** standard deviation of early- and late-phase synaptic weight, demonstrating the matching of the stochastic properties of the two solvers. **(e)** Postsynaptic calcium concentration, which successively crosses the thresholds for depression (LTD) and potentiation (LTP) (cf. Eq. Eq. 19 in the main article). **(f)** The postsynaptic PRP concentration rises until it reaches its maximum due to the continued stimulation (cf. Eq. 21 in the main article). **(g)** Membrane potential of the postsynaptic neuron. Continuous lines (specified in the legends) represent the dynamics simulated in Arbor. Darker, dashed lines represent the results of [2], using Brian 2 with `cpp_standalone` device [3]. Fine, dotted lines represent the baseline level of the respective quantity. Basic early-phase plasticity dynamics (a,c,e,g): average across 10 batches, each consisting of 100 trials. Basic late-phase plasticity dynamics (b,d,f): average across 10 batches, each consisting of 10 trials. The noise seeds were drawn independently for each trial. Error bands represent the standard error of the mean (often too small to be visible).

Simulator cross-validation – details at the network level

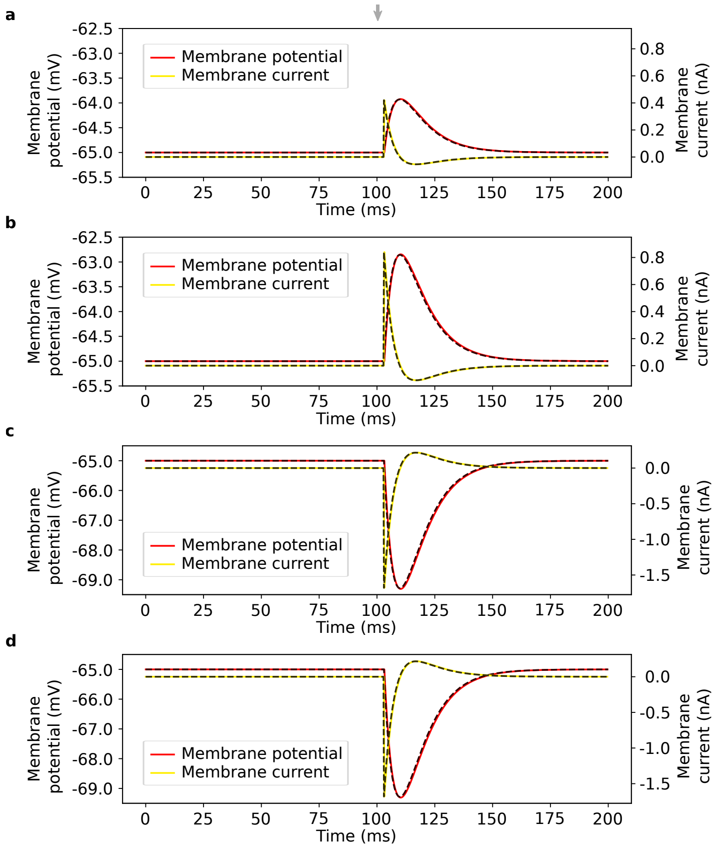

Fig D: **Elementary motifs of spike transmission in a pre-defined sparsely connected network, cross-validated with stand-alone simulator**. Continuous lines (specified in the legends) represent the dynamics simulated in Arbor. Darker dashed lines represent the results of the stand-alone simulator [1]. Stochastic variables in the model have been replaced by deterministic mean dynamics ($\sigma_{\mathrm{pl}} = 0$). The connectivity matrix `connections_default.txt` from [1] is used. **(a)** Response of neuron 68 to a spike in neuron 6 (excitatory→excitatory); **(b)** response of neuron 1760 to a spike in neuron 6 (excitatory→inhibitory); **(c)** response of neuron 17 to a spike in neuron 1615 (inhibitory→excitatory); **(d)** response of neuron 1690 to a spike in neuron 1615 (inhibitory→inhibitory).

Technical details of the implementation of the synaptic tagging and capture model

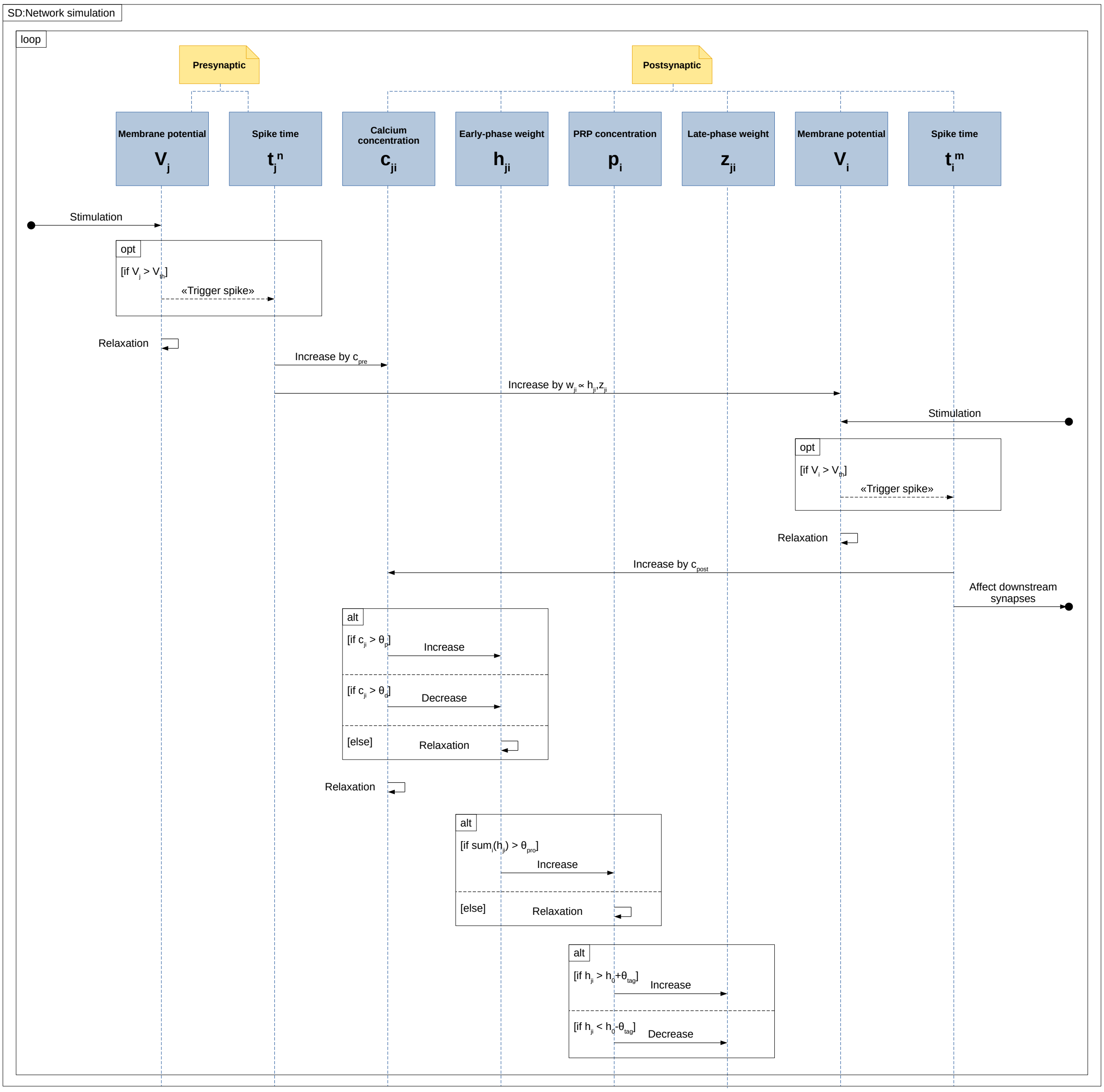

Fig E: **UML sequence diagram for the single-compartment synaptic tagging and capture model.** The diagram describes the technical specification that is necessary to implement the model from [4]. Essentially, the spiking neuron model produces pre- and postsynaptic spikes that give rise to calcium-based early-phase plasticity, which then elicits synaptic tagging, synthesis of plasticity-related products (PRPs), and late-phase plasticity.

Classical protocols for early- and late-phase synaptic plasticity

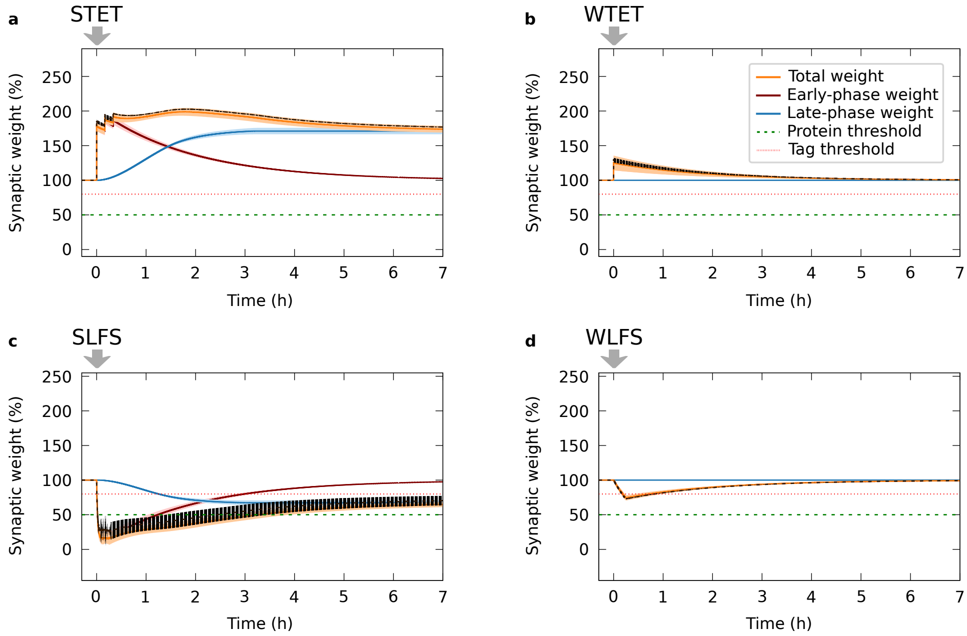

Fig F: **Impact of classical stimulation protocols at a single synapse.** Different types of long-term synaptic plasticity are induced depending on the stimulation protocol [5, 6]: **(a)** late-phase potentiation through strong tetanic (STET) stimulation, **(b)** early-phase potentiation through weak tetanic (WTET) stimulation, **(c)** late-phase depression through strong low-frequency (SLFS) stimulation, **(d)** early-phase depression through weak low-frequency (WLFS) stimulation. See [4] for details of the protocols. All protocols affect the early-phase weight (dark red lines) and lead to the crossing of the tag threshold (dotted red lines), whereas only the 'strong' protocols lead to the crossing of the PRP synthesis threshold (dashed green lines), thereby enabling changes in late-phase weight (blue lines). The total synaptic weight (orange lines) is the sum of early- and late-phase weight. These results were obtained with Arbor using the code from [7] (which has the same basis as our network code [8, 9]). In addition, for comparison, the total synaptic weight obtained from point-neuron simulations with the stand-alone simulator [1] is shown in grated dark shading (overlapping with the total weight from Arbor simulations). Average over 100 trials; sampling rate: 30/min. Compartment measures in Arbor as detailed in Table 5 in the main article. Error bands represent the relative standard deviation of early-phase, late-phase, and total synaptic weight. The late-phase weight has further been shifted for graphical reasons (cf. Eq. 20 in the main article).

Diffusion along dendrites

a

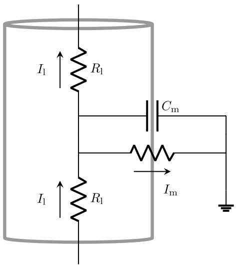

b

**Variable**	**Symbol**
Axial resistance	R_l
Membrane potential	U_m
Membrane capacity	C_m
Radial current	I_m
Axial current	$I_l = \partial_x \frac{1}{R_l} \partial_x U_m$
Internal/external concentration	X_i / X_o
Ion diffusivity	D
Ionic charge	q
Axial ion flux	ϕ

Fig G: **Formal characteristics of the diffusion along a dendritic cable in Arbor.** **(a)** A single compartment and its equivalent circuit in the cable model. **(b)** Important quantities of the diffusion mechanisms.

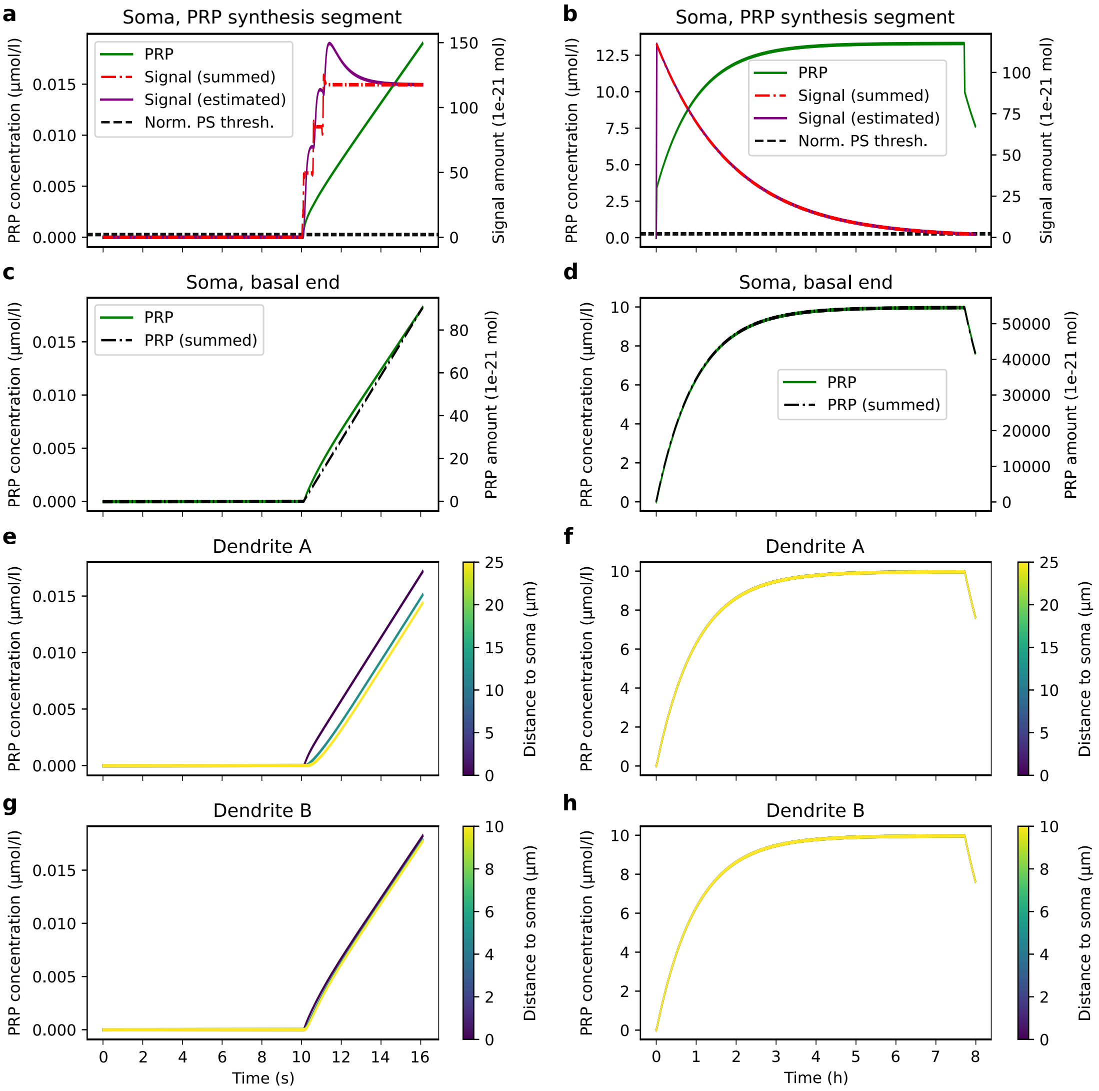

Fig H: **Diffusion of arbitrary particles along a dendrite in Arbor at different times (maximum diffusivity).** Example for paradigm of large dendrites, small cells, and maximum diffusivity $D_\mathrm{p} = 10^{-11}\,\mathrm{m}^2/\mathrm{s}$ (cf. Table 6 in the main article). **(a,b)** Plasticity-related product or protein (PRP) concentration in the center of the soma, and summed amount of the signal triggering PRP synthesis (SPS) across the whole cell. Furthermore, the amount of SPS in the whole cell is estimated from the concentration of SPS in the center of the soma, which is the actual driver of PRP synthesis (PS) in the model. As long as this quantity is above the PS threshold, PS happens (cf. Eqs. 21 & 23 in the main article). **(c,d)** PRP concentration at the basal end of the soma, and summed amount of PRP across the whole cell (under ongoing PS, converges towards p_max times the total volume of the cell). **(e,f)** PRP concentration along apical dendrite. **(g,h)** PRP concentration along basal dendrite.

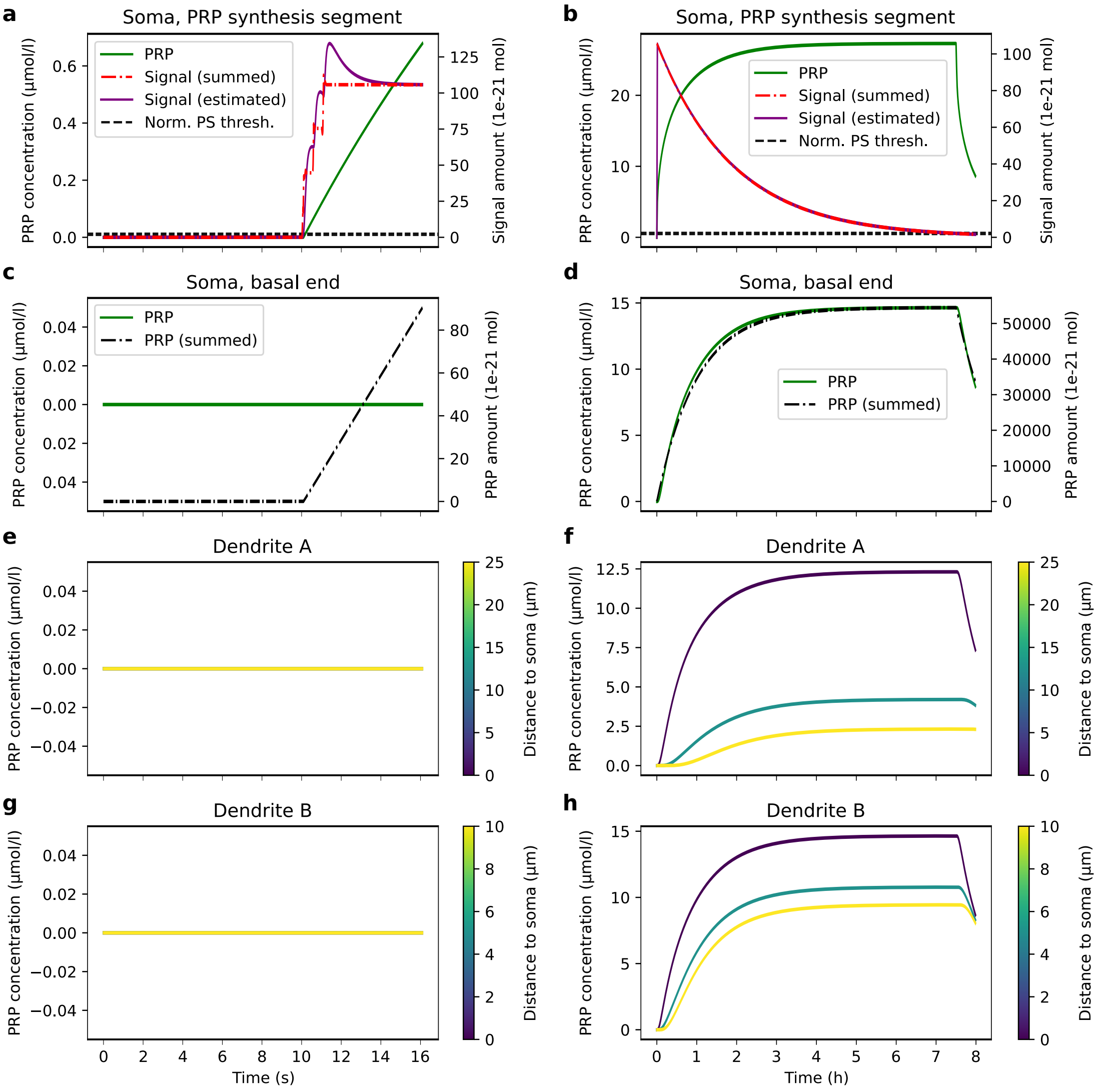

Fig I: **Diffusion of arbitrary particles along a dendrite in Arbor at different times (moderate diffusivity).** Example for paradigm of large dendrites, small cells, and moderate diffusivity $D_\mathrm{p} = 10^{-15}\,\mathrm{m}^2/\mathrm{s}$ (cf. Table 6 in the main article). **(a,b)** Plasticity-related product or protein (PRP) concentration in the center of the soma, and summed amount of the signal triggering PRP synthesis (SPS) across the whole cell. Furthermore, the amount of SPS in the whole cell is estimated from the concentration of SPS in the center of the soma, which is the actual driver of PRP synthesis (PS) in the model. As long as this quantity is above the PS threshold, PS happens (cf. Eqs. 21 & 23 in the main article). **(c,d)** PRP concentration at the basal end of the soma, and summed amount of PRP across the whole cell (under ongoing PS, converges towards p_max times the total volume of the cell). **(e,f)** PRP concentration along apical dendrite. **(g,h)** PRP concentration along basal dendrite.

Synaptic memory consolidation in networks of single-compartment and point neurons (across simulators)

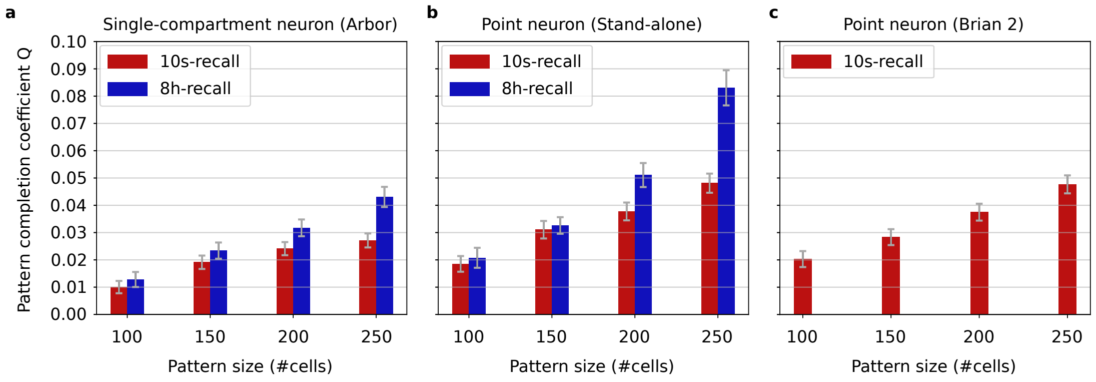

Fig J: **Memory recall in recurrent networks of single-compartment neurons and point neurons, after learning and after consolidation.** Plots show the pattern completion, measured by coefficient Q (see Eq. 22 in the main article) for a stimulated subset of varied size (a varied number of neurons are stimulated for learning/recall). Values at 10 s are shown in red and values at 8 h in blue (after learning and after consolidation, respectively). Value $Q > 0$ indicates the successful recall of a memory representation. Results in **(a)** obtained with Arbor, in **(b)** from the custom stand-alone simulator [1], and in **(c)** from Brian 2 with `cpp_standalone` device [10] (no data for 8 h due to missing fast-forward computation implementation). Data averaged over 100 network realizations (unlike in [4]). Error bars represent the 95% confidence interval.

Synaptic memory consolidation in networks of morphological neurons – mutual information

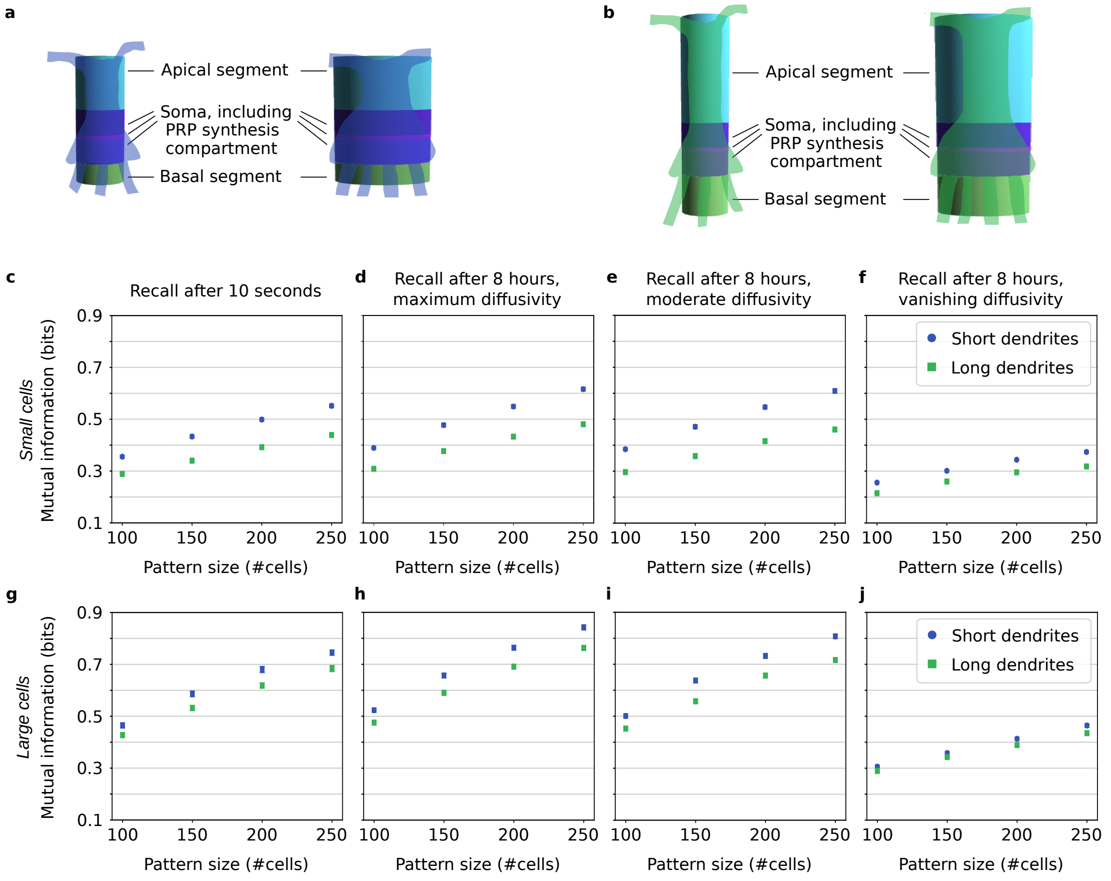

Fig K: **Memory recall in a recurrent neural network after learning and after consolidation.** Analogous to Fig 5 in the main article, a measure of mutual information is considered here. Results obtained with Arbor for networks of different kinds of multi-compartment neurons, demonstrating the impact of different values of the PRP diffusivity D_p on memory consolidation. Networks consist of 'small' cells (radius of 6 µm) or of 'large' cells (radius of 12 µm), with either short or long dendrites (in which cases each neuron comprises in total 31 or 48 compartments, respectively). The radius and length values are given in Table 6 in the main article. **(a,b)** Illustrations of used cell structures, generated using `Arbor GUI` [11]. Each segment is represented by a different color. A segment can consist of a multitude of compartments. Overlaid with illustrations of more realistic neuron structures that would have roughly similar functional properties. **(a)** Small (left) and large (right) cell with short dendrites, **(b)** the same with long dendrites. **(c-f)** Memory recall measured by the mutual information between the distribution of neuronal firing rates during learning and during recall stimulation (see [4]), for a stimulated subset of varied size (a varied number of neurons are stimulated for learning/recall). Average over 100 network realizations. Error bars represent the 95% confidence interval. **(c)** Recall stimulation at 10 s after learning (technically, $D_\mathrm{p} = 10^{-11}\,\mathrm{m}^2/\mathrm{s}$, but late-phase plasticity does not occur at such fast timescales). **(d)** Recall stimulation at 8 h after learning, $D_\mathrm{p} = 10^{-11}\,\mathrm{m}^2/\mathrm{s}$. **(e)** Recall stimulation at 8 h after learning, $D_\mathrm{p} = 10^{-15}\,\mathrm{m}^2/\mathrm{s}$. **(f)** Recall stimulation at 8 h after learning, $D_\mathrm{p} = 10^{-19}\,\mathrm{m}^2/\mathrm{s}$. **(g-j)** Same as (c-f), but for large cells that consist of segments of twice the diameter.

Runtime and memory benchmarking with the synaptic memory consolidation model – additional data

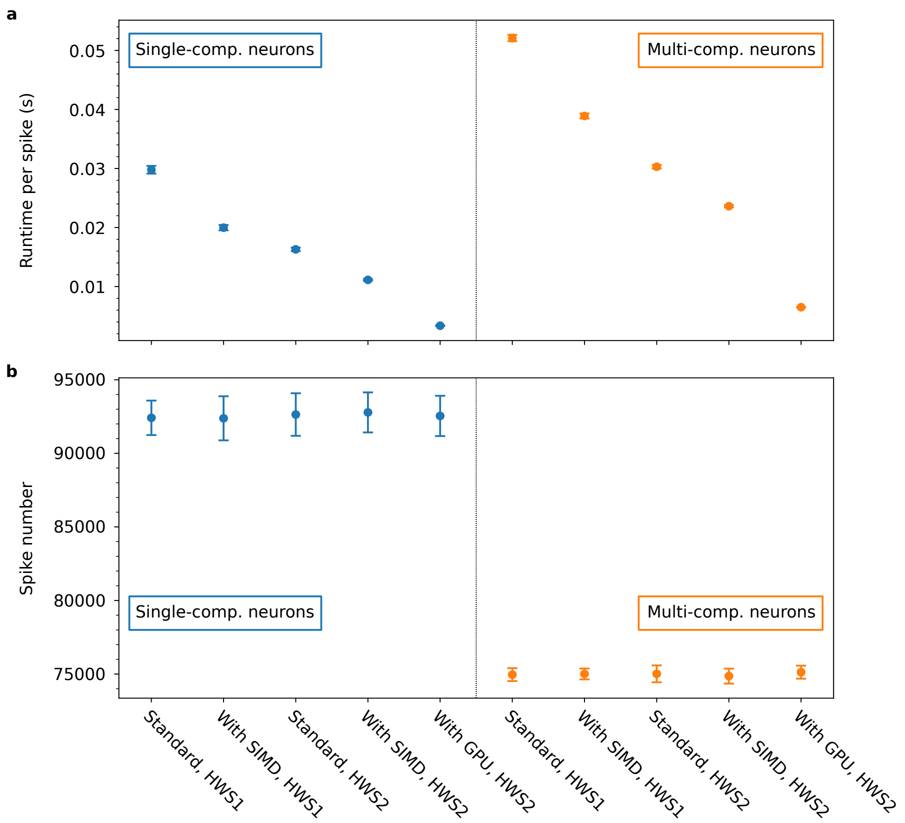

Fig L: **Benchmarking results of total runtime per spike**, for 10s-recall paradigm in networks of 2000 neurons, related to Arbor simulations in Fig 6 in the main article. **(a)** Total runtime (including setup and propagation phase) per spike, accounting for the fact that the spike numbers in the single-compartment and multi-compartment simulations are different (cf. panel (b)). The data points show the runtime as given in Fig 6 in the main article, divided by the total number of spikes. **(b)** Total number of spikes in the different simulation paradigms. Data points represent the average over 10 trials; error bars represent the standard deviation.

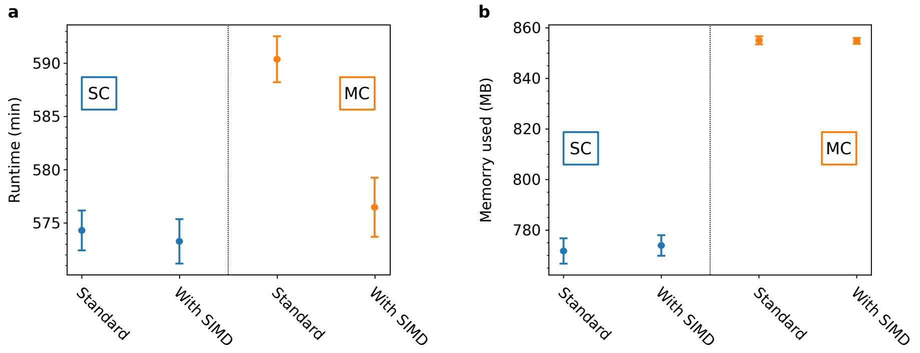

Fig M: **Benchmarking results of total runtime and memory use for 8h-recall paradigm.** Networks of 2000 neurons (also see Fig 6 in the main article) in Arbor. The single-compartment simulations ('SC') are conducted as described in subection 3.5 in the main article. The simulations with multi-compartment/morphological neurons ('MC') are conducted as described in subsection 3.6. Results are given for execution with and without SIMD support on the HWS1 system (Intel Core i5-6600 CPU @ 3.30GHz, 1×8 GB DDR3-RAM, using 1 thread). **(a)** Total runtime of the simulations, including setup and propagation phase. Measurements were performed using `hyperfine` in version 1.15. **(b)** Memory consumption, given by the maximum over time of the number of 'dirty' bytes, including private and shared memory, as returned from the `pmap` tool. Data points represent the average over 10 trials; error bars represent the standard deviation.

Runtime and memory benchmarking with large-scale networks – additional data

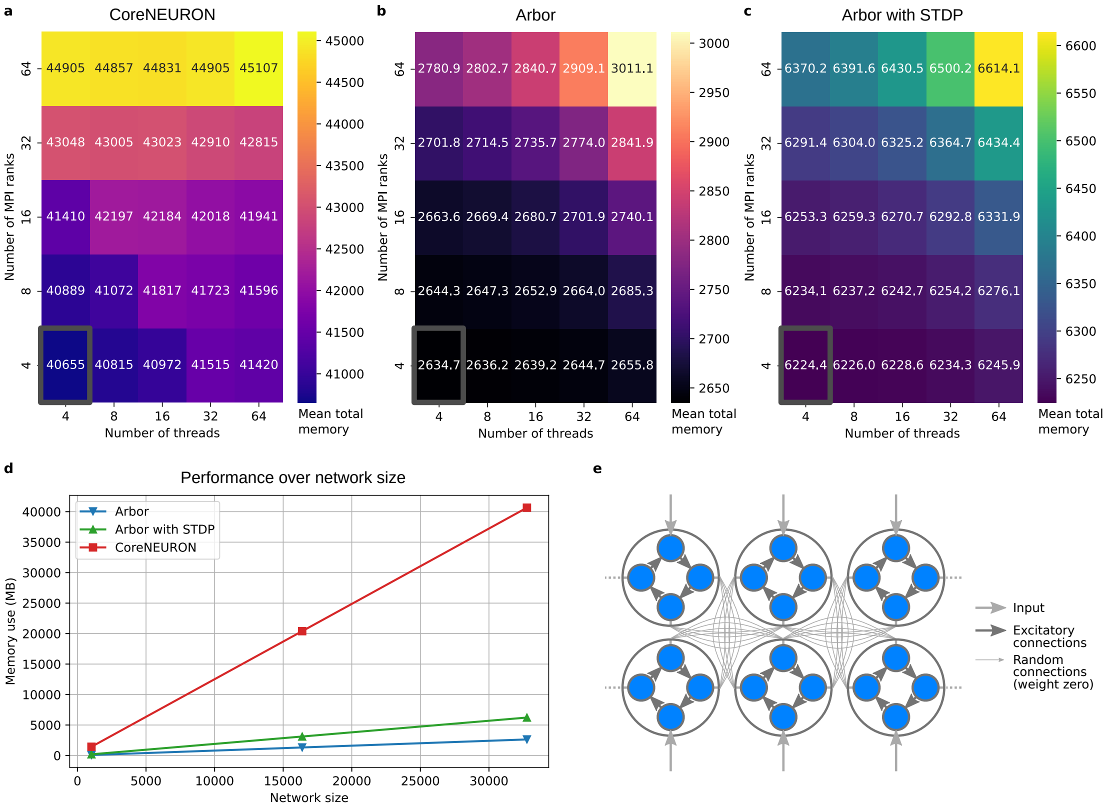

Fig N: **Benchmarking of total memory use for large-scale networks.** Total memory use, in megabytes, to initialize and execute a simulation with 32768 cells over 200 ms in Arbor and CoreNEURON. A busyring network of `simple-branchy` cells with tree depth 2 is used, run on the HWS2 system (AMD Ryzen Threadripper PRO 5995WX CPU with 64 cores, 8 × 32 GB DDR4-RAM) with **(a)** CoreNEURON, **(b)** Arbor with SIMD, **(c)** Arbor with SIMD with additional STDP mechanisms for the random synapses. The respectively lowest memory use for each implementation is highlighted by the gray box. **(d)** Scaling of the best results over network size. **(e)** Sketch of the busyring network consisting of rings of integrate-and-fire neurons (shown as blue disks), connected internally via excitatory synapses, and across the whole network via random synapses of weight zero. One neuron of each ring receives external stimulation. All values are averaged over 10 trials, with coefficient of variation CV < 0.004 in all cases. See Table A for more details.

Number of cells	Tree depth	Number of synapses	Number of compartments	Memory use of CoreNEURON (MB)	Memory use of Arbor (MB)	Memory use of Arbor with STDP (MB)
1024	0	1.03M	1024	1387	72	186
	2	1.03M	46116	1432	81	193
	10	1.03M	1672700	2520	336	420
16384	0	16.41M	16384	19924	1163	2987
	2	16.41M	737408	20398	1318	3113
	10	16.41M	26616896	39819	5338	6686
32768	0	32.81M	32768	39662	2326	5974
	2	32.81M	1475268	40654	2634	6224
	10	32.81M	52957704	d.n.f.	10681	13377

Table A: **Total memory use for busyring benchmark.** Measurements are provided for networks of `simple-branchy` cells as reported by Arbor and CoreNEURON (using the lowest-memory paradigm as detailed in Fig N). Results are collected with the HWS2 system (AMD Ryzen Threadripper PRO 5995WX CPU with 64 cores, 8×32 GB DDR4-RAM, not using the GPU). All values are averaged over 10 trials, with coefficient of variation CV < 0.004 in all cases. Arbor with SIMD. In CoreNEURON, the most extensive simulation did not finish (d.n.f.) due to exceeded memory. For the corresponding runtime results, cf. Table 8 in the main article.

Cell type	Tree depth	Number of compartments	Runtime of Arbor (s)	Runtime of Arbor with STDP (s)
`simple-branchy`	0	1024	0.23 (0.04 + 0.19)	0.72 (0.05 + 0.67)
`simple-branchy`	2	46116	0.32 (0.04 + 0.28)	0.83 (0.05 + 0.78)
`simple-branchy`	10	1672700	2.67 (0.19 + 2.48)	3.22 (0.21 + 3.01)
`complex`	0	1024	0.51 (0.05 + 0.47)	1.07 (0.06 + 1.02)
`complex`	2	46116	1.07 (0.08 + 0.99)	1.62 (0.09 + 1.53)
`complex`	10	1672700	23.34 (0.50 + 22.84)	23.95 (0.53 + 23.43)

Table B: **Wallclock time measurements for busyring benchmark with different cell types.** Total runtime results are provided as reported by Arbor (using the fastest paradigm as detailed in Fig 7B,C in the main article). All networks consist of 1024 neurons and 1.03 million synapses. The shares of the setup and state propagation phases are given in brackets, respectively. Results are collected with the HWS2 system (AMD Ryzen Threadripper PRO 5995WX CPU with 64 cores, 8×32 GB DDR4-RAM, not using the GPU). All values are averaged over 10 trials, with coefficient of variation CV < 0.06 in all cases. Arbor with SIMD. Also see Tables 8 and 9 in the main article for other paradigms.

Property	JUWELS	JEDI
Node count	8–64	4–16
Ranks per node	4	4
Threads/rank	12	12
CPU type	Xeon 6148	GH200
GPU model	4 × A100	4 × H100
Number of cells	10^6	10^6
Compartments per cell	85	85

Table C: **Parameters and hardware characteristics for investigating strong scaling on supercomputing systems.** We use one MPI rank per GPU and the full set of 4 GPUs per node when present in the node architecture. See Table 7 in the main article for further parameter values.

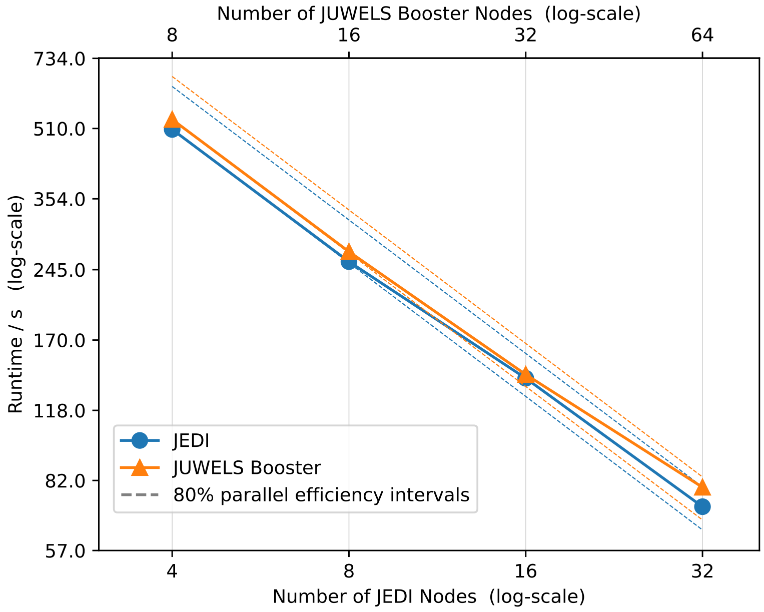

Fig O: **Strong scaling on the JUWELS and JEDI supercomputing systems.** Benchmarking results for the total wallclock time, including setup and state propagation phases, of a simulation with 10^6 `complex` cells over 200 ms. We show strong scaling, i.e., the cell count is subdivided across the available GPUs. For reference, the intervals of 80% efficiency are shown, where 100% efficiency represents perfect scaling with the additional hardware. The data was obtained during the pre-production phase of the JUPITER supercomputing system on the JEDI preview system. Parameter values are provided in Table 7 in the main article; further parameters and hardware characteristics are provided in Table C.

References

[1] Luboeinski, J. & Lehr, A. Simulation code and analysis scripts for memory formation and consolidation with synaptic tagging and capture in recurrent spiking neural networks (2024). URL https://doi.org/10.5281/zenodo.4429195.

[2] Luboeinski, J. Brian 2 simulation of the induction of early- and late-phase plasticity at a single synapse (2023). URL https://github.com/jlubo/brian_synaptic_plasticity_stc. Accessed: 23 November 2024.

[3] Stimberg, M., Brette, R. & Goodman, D. F. Brian 2, an intuitive and efficient neural simulator. *eLife* **8**, e47314 (2019).

[4] Luboeinski, J. & Tetzlaff, C. Memory consolidation and improvement by synaptic tagging and capture in recurrent neural networks. *Communications Biology* **4**, 275 (2021).

[5] Sajikumar, S., Navakkode, S., Sacktor, T. C. & Frey, J. U. Synaptic tagging and cross-tagging: the role of protein kinase Mζ in maintaining long-term potentiation but not long-term depression. *Journal of Neuroscience* **25**, 5750–5756 (2005).

[6] Li, Y., Kulvicius, T. & Tetzlaff, C. Induction and consolidation of calcium-based homo- and heterosynaptic potentiation and depression. *PLOS One* **11**, e0161679 (2016).

[7] Luboeinski, J. Arbor simulation of the induction of early- and late-phase plasticity at a single synapse (2023). URL https://github.com/jlubo/arbor_2N1S. Accessed: 23 November 2024.

[8] Luboeinski, J. Arbor simulation of memory formation and consolidation in recurrent spiking neural networks with synaptic tagging and capture (2024). URL https://github.com/jlubo/arbor_network_consolidation. Accessed: 17 December 2024.

[9] Luboeinski, J. Arbor simulation of memory formation and consolidation in recurrent networks of spiking multi-compartment neurons with synaptic tagging and capture (2024). URL https://github.com/jlubo/arbor_network_consolidation_mc. Accessed: 17 December 2024.

[10] Luboeinski, J. Brian 2 simulation of memory formation and consolidation in recurrent spiking neural networks based on synaptic tagging and capture (2024). URL https://github.com/jlubo/brian_network_plasticity. Accessed: 23 November 2024.

[11] Hater, T. *et al.* Arbor GUI v0.8 (2022). URL https://doi.org/10.5281/zenodo.7415130.
